# TIPE3 protein promotes breast cancer metastasis through activating AKT and NF-κB signaling pathways

**DOI:** 10.18632/oncotarget.16522

**Published:** 2017-03-23

**Authors:** Kaili Lian, Chao Ma, Chunyan Hao, Yan Li, Na Zhang, Youhai H. Chen, Suxia Liu

**Affiliations:** ^1^ Department of Immunology, Shandong University School of Medicine, Ji'nan, P.R. China; ^2^ Department of Pathology, Shandong University School of Medicine, Ji'nan, P.R. China; ^3^ Department of Pathology and Laboratory Medicine, University of Pennsylvania, Philadelphia, PA, USA

**Keywords:** TNFAIP8L3, breast cancer, metastasis, AKT, NF-κB

## Abstract

TIPE3 (TNFAIP8L3) is the transfer protein of phosphoinositide second messengers that promote cancer. Its role in breast cancer has not been evaluated. We report here that TIPE3 protein was significantly upregulated in human breast cancer tissues as compared with adjacent non-tumor tissues from the same patients. The level of TIPE3 protein in invasive ductal carcinoma was significant higher than that in ductal carcinoma *in situ* (DCIS), and the level of TIPE3 in lymphatic metastasized carcinoma was higher than that in invasive ductal carcinoma from the same patients. Additionally, the level of TIPE3 protein was positively correlated with the level of human epidermal growth factor receptor 2 (HER-2), and TIPE3 expression was significantly higher in high-invasive breast cancer cell lines than that in low-invasive cell lines. Importantly, TIPE3 knockdown in breast cancer cells inhibited cell proliferation, migration, and invasion *in vitro*, whereas TIPE3 overexpression had the opposite effect. In mice, TIPE3 expression significantly promoted the metastasis of breast cancer cells. TIPE3 expression also increased the level of MMP2 and uPA, and the activation of the AKT and NF-κB signaling pathways. These results demonstrate that TIPE3 may promote breast cancer growth and metastasis through AKT and NF-κB, and may serve as a potential biomarker for breast cancer metastasis.

## INTRODUCTION

Breast cancer is a leading cause of cancer death among women worldwide, particularly in Western countries [1]. In China, breast cancer accounts for 12.2% of all newly diagnosed cancers and 9.6% of all death [2]. The majority of breast cancer-related deaths are caused by distant metastases, which is due to the spread of cancer cells from the primary location to new organ sites through blood capillaries or draining lymphatic vessels [3, 4]. The prognostic diagnosis of human breast cancer still depends on conventional pathologic variables, such as pathological markers, lymph node metastasis, and tumor size [5]. Therefore, it is of great clinical value to identify molecules that can regulate growth and metastasis, and serve as early biomarkers for the diagnosis and treatment of the disease.

TIPE3, tumor necrosis factor-alpha-induced protein 8 (TNFAIP8)-like 3 (TNFAIP8L3), is a newly identified protein of the TNFAIP8 (TIPE) family [6]. The mammalian TNFAIP8 family consists of four members: TIPE, the first identified member of this family, TIPE1, TIPE2, and TIPE3, which share high degrees of sequence homology and are involved in oncogenic transformation, metastasis, inflammation and cell death [6–9]. In recent years, most studies have focused on TIPE2, which negatively regulates both carcinogenesis and inflammation [6, 10–16]. Among these family members, TIPE is known as an apoptosis regulator and is thought to play an important role in carcinogenesis and tumor metastasis [17–20]. TIPE also plays an important role in maintaining colon homeostasis and in protecting against colitis [21]. It's reported that TIPE1 was a necessary component of both zVAD-fmk caspase inhibitor and TNF-α-induced necroptosis [8, 22, 23]. TIPE1 has been reported to induce cell death and negatively regulates Rac1 activation in hepatocellular carcinoma cells [23, 24].

Recent studies show that TIPE3 is expressed in several human organs and is highly upregulated in several human carcinomas including lung cancer, esophageal cancer, cervical cancer, colon adenocarcinoma, and so on [25, 26]. These data suggest that TIPE3 may be involved in tumorigenesis. Crystal structure of TIPE3 shows a large hydrophobic cavity that can carry lipid second messengers such as PtdIns(4,5)P2 or PtdIns(3,4,5)P3. TIPE3 can induce cancer cell malignant transformation by promoting the activation of the PI3K-AKT pathway [26]. However, the role of TIPE3 in human breast cancer remains to be established. We report here that human TIPE3 could promote proliferation, invasion, and metastasis of breast cancer cells, and may serve as a potential biomarker and therapeutic target for advanced breast cancer.

## RESULTS

### The upregulation of TIPE3 protein in human breast cancer was positively associated with cancer metastasis

**Figure 1:**
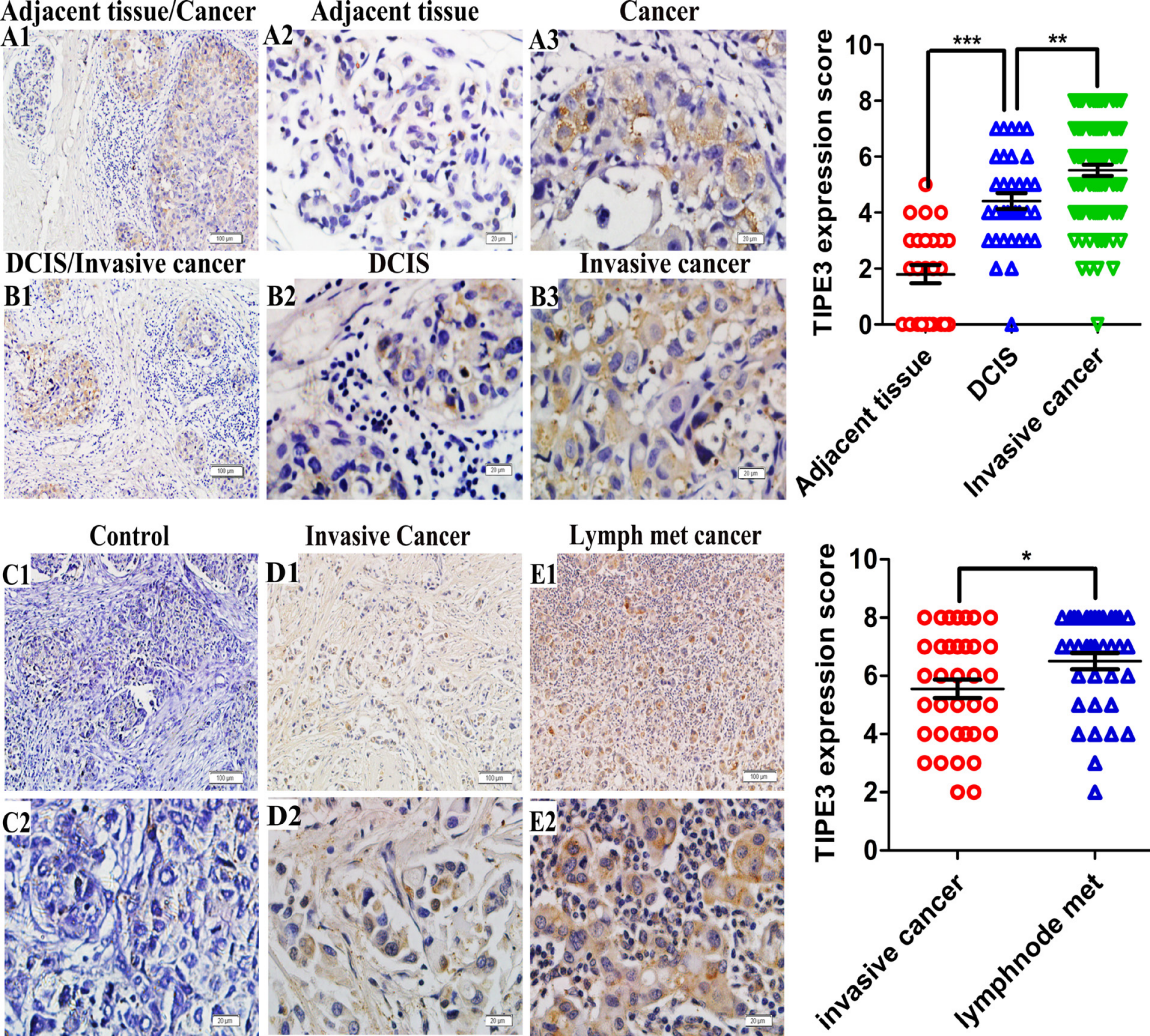
TIPE3 expression in breast cancer as determined by IHC **A1** and **A2** showed TIPE3 expression in adjacent breast tissue (A1, 100 ×; A2, 400 ×). TIPE3 protein was detected in ductal carcinoma *in situ* (DCIS) and invasive ductal carcinoma (**B1**, 100 ×; **B2**, B3 and A3, 400 ×). **D1** and **D2** showed TIPE3 expression in invasive ductal carcinoma (D1, 100 ×; D2, 400 ×). **E1** and **E2** showed TIPE3 expression in lymphatic metastasis carcinoma (**E1**, 100 ×; **E2**, 400 ×). Isotype control was not staining (**C1**, 100 ×; **C2**, 400 ×). All of the experiments were repeated at least three times with similar results and representative data are shown. (*P < 0.005, **P < 0.001 and ***P < 0.001).

In order to determine the roles of TIPE3 in human breast cancer, we examined the expression of TIPE3 protein in 96 human breast cancer samples and microarrays by immunohistochemistry. We found that TIPE3 was significantly upregulated in the majority of tumor sections compared with adjacent non-tumor section from the same patients (Figure 1A1–1A3, Supplementary Table 1, Table 1, *P* < 0.001). Importantly, the staining of TIPE3 protein in invasive ductal carcinoma was stronger than in DCIS from the same patients (Figure 1B1–1B3, Supplementary Table 1, Table 1, *P* = 0.007). Furthermore, TIPE3 expression in the lymphatic metastasized carcinoma was stronger than that in invasive ductal carcinoma from the same patients (Figure 1D1 and 1D2, 1E1 and 1E2, Supplementary Table 2, Table 2, *P* = 0.032). Isotype control staining showed no cross-reactivity (Figure 1C1 and 1C2). These results suggested that the upregulation of TIPE3 protein might be associated with the carcinogenesis and metastasis of human breast cancer. Analysis of clinical pathological features of human breast cancer confirmed this notion. As shown in Table 1, the expression of TIPE3 was positively correlated with the HER2 expression (*P* = 0.024, *r* = 0.325), but there was no significant correlation between TIPE3 expression and age, tumor size, ER status, or PR status (*P* > 0.05). In addition, we also found that the expression level of TIPE3 is higher in HER2-positive breast cancer cells (SKBR3) than that in HER2-negative breast cancer cells (MCF-7 and MDA-MB-231) (Supplementary Figure 1A) [27–29]. These data indicate that the expression of TIPE3 was positively correlated with the metastatic potential of human breast cancer.

**Table 1:** Relationship between TIPE3 expression in breast cancer and clinical pathological features of patients

Clinicalpathologicalfeatures	Number ofcases	Expression of TIPE3				*P* -value	*r*
		−	+	++	+++		
Pathology diagnosis							
Adjacent tissues	25	15	9	1	0	< 0.001	
Cancer tissues	96	6	27	31	32		
DCIS*	10	1	5	3	1	0.007	
Invasive cancer with DCIS*	24						
DCIS tissues	24	2	11	7	4		
Invasive cancer tissues	24	1	5	8	10		
Invasive cancer*	62	4	17	20	21		
Her2 status							
Low	29	2	11	9	7	0.024	0.325
High	19	1	2	6	10		
Age at diagnosis							
< 50	49	4	14	16	15	0.851	
≥ 50	47	2	13	15	13		
Tumor size							
≤ 2.0cm	24	2	7	6	9	0.831	
> 2.0cm	24	1	6	9	8		
ER status							
Low	20	1	4	10	5	0.863	
High	28	2	9	5	12		
PR status							
Low	23	1	5	8	9	0.385	
High	25	2	8	7	8		

Note: *P* < 0.001, TIPE3 protein in Adjacent tissues *vs* TIPE3 in Cancer tissues.

P* = 0.007, TIPE3 protein in DCIS tissues *vs* TIPE3 in invasive tissues.

*P* = 0.024, TIPE3 protein in low HER2 status tissues *vs* in high HER2 status tissues.

DCIS: ductal carcinoma *in situ*.

Her2: human epidermal growth factor receptor 2.

ER: estrogen receptor.

PR: progesterone receptor.

**Table 2:** Comparison of TIPE3 expression levels between invasive breast cancer and lymph node metastatic tissues from same patients

Invasive cancer withLymph node metastasis	*N* = 36	Expression of TIPE3				*P*-value
		-	+	++	+++	
Invasive cancer	36	2	10	10	14	0.032
Lymph node metastasis	36	1	5	7	23	

Note: *P* = 0.032, TIPE3 protein in lymphatic metastasis carcinoma *vs* in invasive ductal carcinoma.

### TIPE3 promotes the proliferation of breast cancer cells *in vitro*

**Figure 2:**
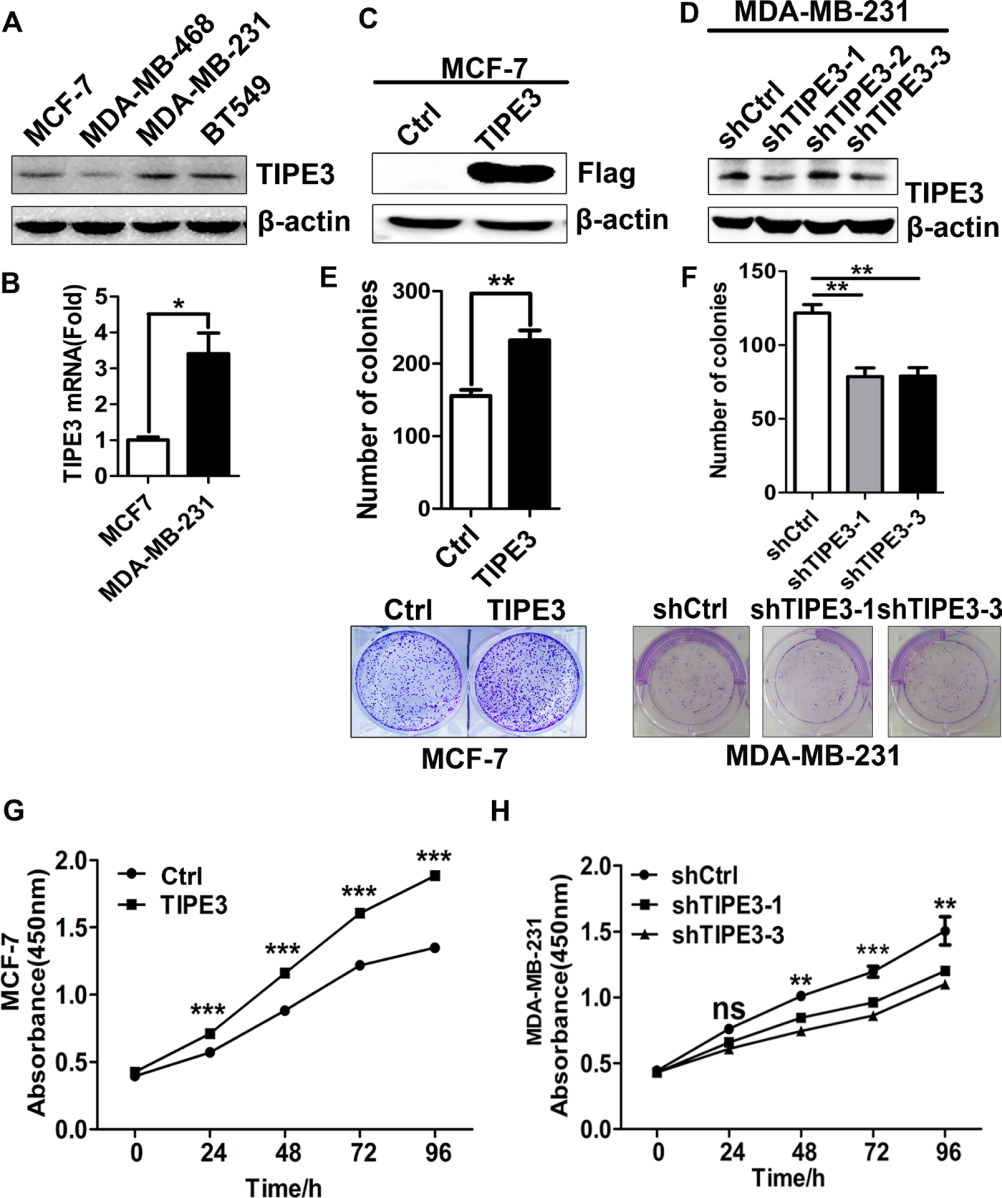
TIPE3 promoted proliferation of human breast cancer cells TIPE3 expression was analyzed by Western blotting (**A**) and qRT-PCR (**B**) in breast cancer cells. Overexpressed TIPE3 in MCF-7 promoted cell proliferation and colony formation (**C**, **E** and **G**). Knocking down TIPE3 in MDA-MB-231 inhibited cells proliferation and colony formation (**D**, **F** and **H**). All of the experiments were repeated at least three times with similar results and representative data are shown. (*P < 0.05, **P < 0.01 and ***P < 0.001).

To further explore the roles of TIPE3 in breast cancer, we next examined TIPE3 expression in several breast cancer cell lines by Western blot and quantitative RT-PCR, and found that the levels of TIPE3 expression were higher in high-invasive cancer cells such as MDA-MB-231 and BT549 than those in low-invasive cancer cells such as MCF-7 and MDA-MB-468 (Figure 2A and 2B). Therefore, TIPE3 expression vector was used to transfect MCF-7 and MDA-MB-231 cells (denoted as MCF-7-TIPE3 and MDA-MB-231-TIPE3, respectively, Figure 2C, Supplementary Figure 2A) to upregulate TIPE3 expression, whereas MDA-MB-231 cells were infected with Lenti-shRNA-hTIPE3 to knock down TIPE3 expression (denoted as MDA-MB-231-shTIPE3, Figure 2D). CCK8 method and colony formation assay were performed to determine the proliferative ability of breast cancer cells. We found that TIPE3 overexpression significantly increased the numbers of MCF-7 and MDA-MB-231 cells and the size of colonies formed in soft agar (Figure 2G and 2E, Supplementary Figure S2B and 2C). Conversely, knocking down TIPE3 in MDA-MB-231 cells significantly reduced cell proliferation (Figure 2H) and clonogenicity (Figure 2F).

### TIPE3 accelerates cell cycle progression

**Figure 3:**
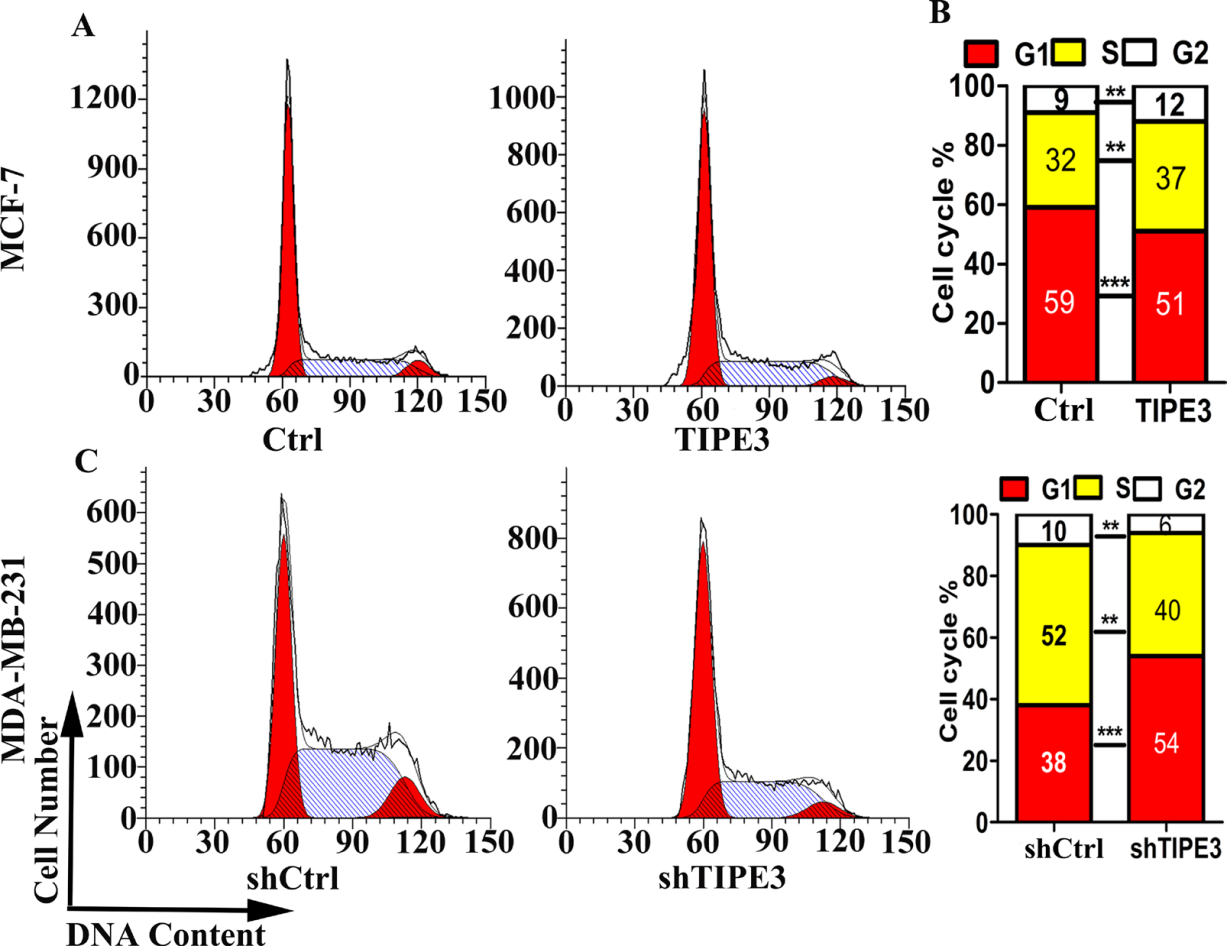
TIPE3 accelerated cell cycle progression of human breast cancer cells Cycle progression of MCF-7 cells transfecting TIPE3 or Ctrl was examined by flow cytometry (**A** and **B**). Cycle progression of MDA-MB-231 cells infecting shTIPE3 or shCtrl was examed by flow cytometry (**C** and **D**). All of the experiments were repeated at least three times with similar results and representative data are shown. (**P < 0.01 and ***P < 0.001).

As shown in Figure 3, overexpression of TIPE3 in MCF-7 cells significantly reduced the population of G_0_/G_1_ cells and increased S/G_2_/M cells (Figure 3A and 3B), whereas knockdown of TIPE3 in MDA-MB-231 cells significantly increased the population of G_0_/G_1_ cells and reduced S/G_2_/M cells (Figure 3C and 3D). These results indicated that TIPE3 accelerated cell cycle progression of human breast cancer cells, suggesting that it could promote breast tumor cell growth.

### TIPE3 markedly promotes tumor cell migration and invasion *in vitro*

**Figure 4:**
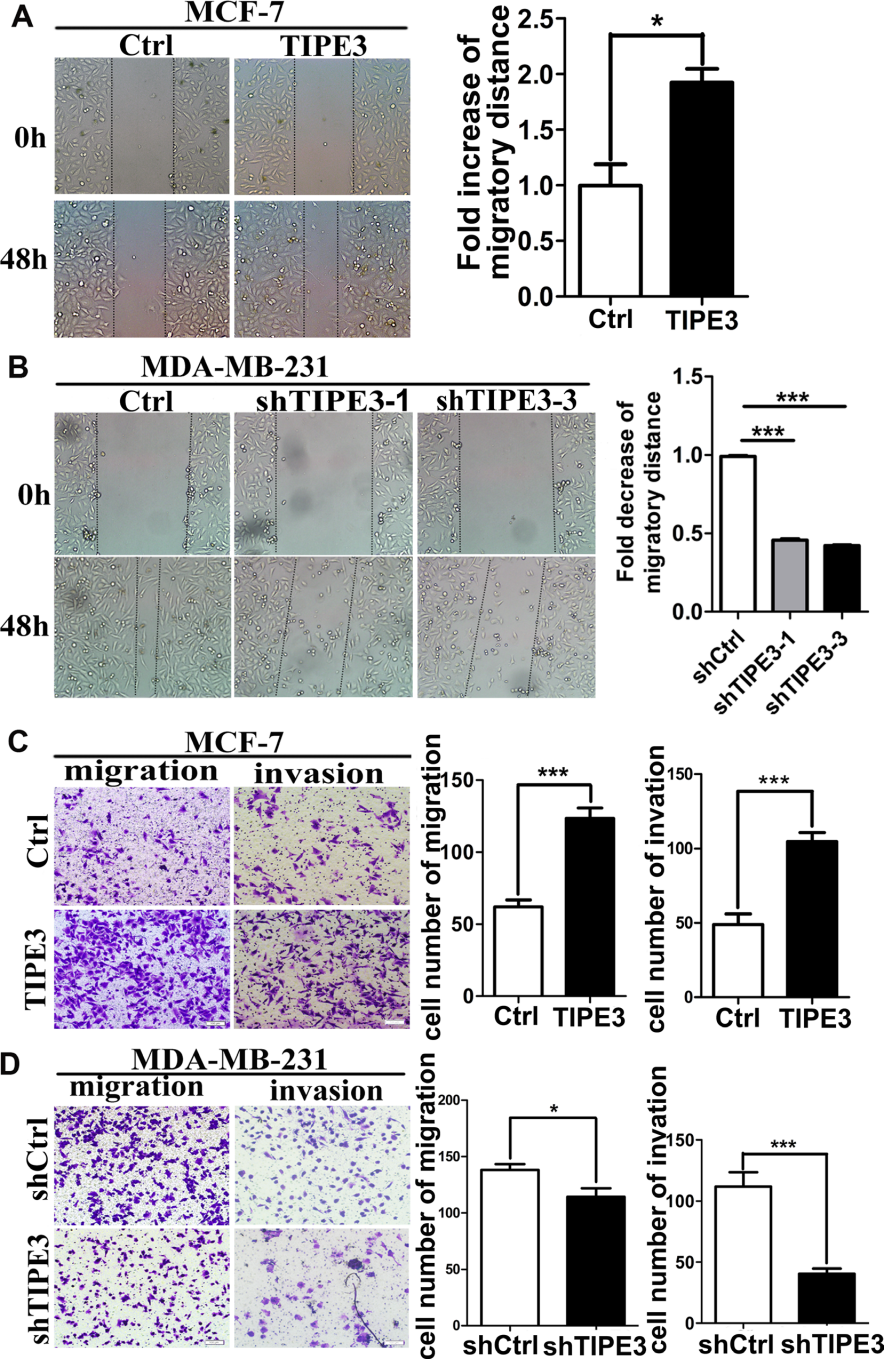
TIPE3 promoted migration and invasion of breast cancer cells MCF-7-pRK5-hTIPE3 and MDA-MB-231-shTIPE3 cells or Ctrl cells were subjected to wound healing assay (**A** and **B**), transwell migration and Matrigel invasion assays (**C** and **D**), respectively. Distance of cells migration was quantified and statistical analysis. Quantification of migrated cells through the membrane and invaded cells through Matrigel of each cell line are statistical analysis. All of the experiments were repeated at least three times with similar results and representative data are shown. (*P < 0.05, **P < 0.01 and ***P < 0.001).

To explore the roles of TIPE3 in cell motility, wound healing assay, transwell migration and matrigel invasion assay were performed. As shown in Figure 4, TIPE3 overexpression in MCF-7 and MDA-MB-231 cells could promote tumor cell migration and invasion markedly (Figure 4A and 4C, Supplementary Figure 3A and 3B). Conversely, knocking down TIPE3 in MDA-MB-231 cells dramatically inhibited the migration and invasion (Figure 4B and 4D). These results indicate that TIPE3 could significantly promote migration and invasion of breast cancer cells.

### TIPE3 promotes proliferation, migration and invasion of breast cancer cells by activating AKT and NF-κB pathways

**Figure 5:**
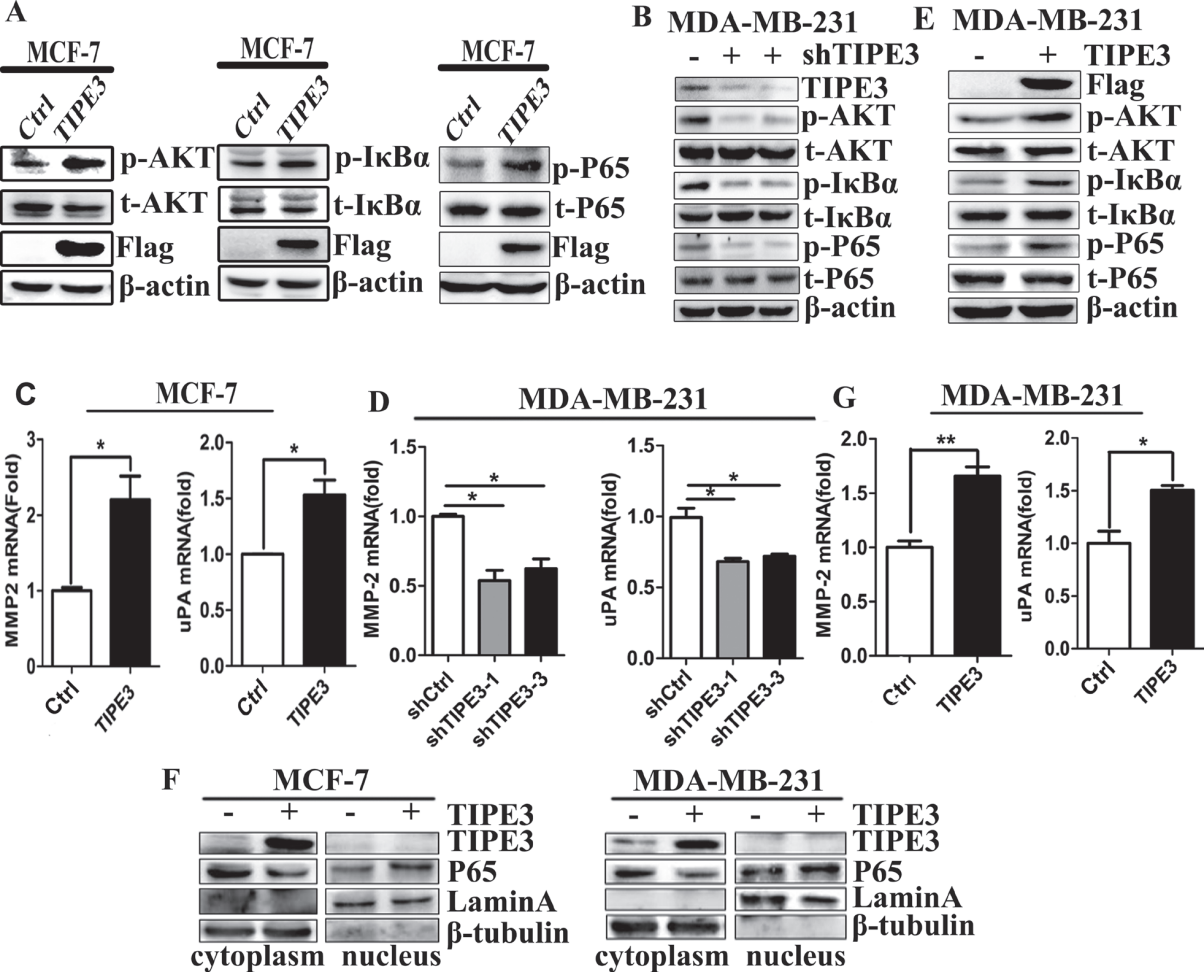
TIPE3 upregulated the expression of MMP2 and uPA, and promoted the activation of AKT and NF-κB The phosphorylation levels of p-AKT, p-IkBα and p-P65 were upregulated by overexpressed TIPE3 in MCF-7 cells (**A**) and MDA-MB-231 cells (**E**), and were downregulated in MDA-MB-231 cells transfected with shTIPE3 (**B**). The levels of MMP2 and uPA were increased in MCF-7-TIPE3 (**C**) and MDA-MB-231-TIPE3 (**G**) cells, and were decreased in MDA-MB-231-shTIPE3 cells (**D**). The levels of nuclear P65 were upregulated in TIPE3-overexpressed MCF-7 and MDA-MB-231 cells (**F**). All of the experiments were repeated at least three times with similar results and representative data are shown. (*P < 0.05, **P < 0.01 and ***P < 0.001).

As shown in Figure 5, TIPE3 overexpression in MCF-7 and MDA-MB-231 cells substantially increased the levels of AKT, IkBα and P65 phosphorylation (Figure 5A and 5E), while TIPE3 knockdown in MDA-MB-231 cells decreased their phosphorylation levels (Figure 5B and Supplementary Figure 4). As expected, overexpressed TIPE3 in MCF-7 or MDA-MB-231 cells promoted p65 nuclear localization (Figure 5F). To further explore the mechanisms involved, we examined the expression of migration-related molecules including MMP-2 and uPA by qRT-PCR. Compared with controls, the levels of MMP-2 and uPA were significantly upregulated in MCF-7-TIPE3 and MDA-MB-231-TIPE3 cells with TIPE3 overexpression (Figure 5C and 5G). Conversely, TIPE3 knockdown in MDA-MB-231 cells inhibited the expression of MMP-2 and uPA molecules (Figure 5D).

**Figure 6:**
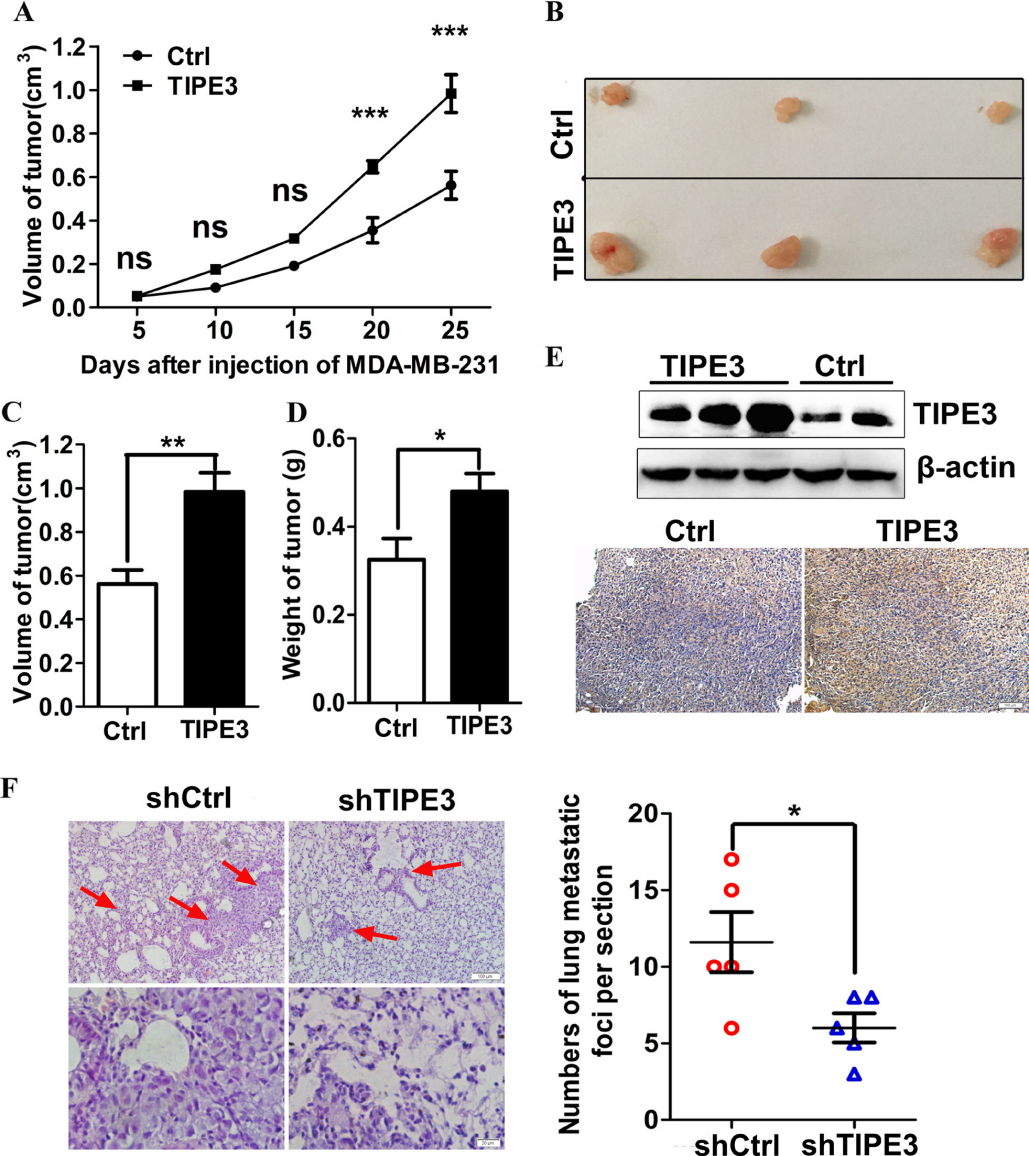
TIPE3 promotes tumorigenesis and metastasis of human breast cancer *in vivo* Compared with the group injected with pRK5-Ctrl, the numbers of nude mice appearing tumors are more in the group injected with pRK5-TIPE3. The growth curves of tumors in nude mice injected with pRK5-Ctrl and pRK5-TIPE3 plasmid (**A**). (**B**) showed the representative image of isolated tumors. The mean tumor volume and weight in TIPE3 group (*n* =7) was higher than that of pRK5-Ctrl group (*n* =7) (**C** and **D**). The expression of TIPE3 in tumor tissues was detected by WB and immunohistochemistry (**E**). The numbers of metastatic foci per section in lung of individual nude mice with injection of MDA-MB-231-shTIPE3 or its shCtrl cells (**F**). All of the experiments were repeated at least three times with similar results and representative data are shown. (*P < 0.05, **P < 0.01 and ***P < 0.001).

**Figure 7:**
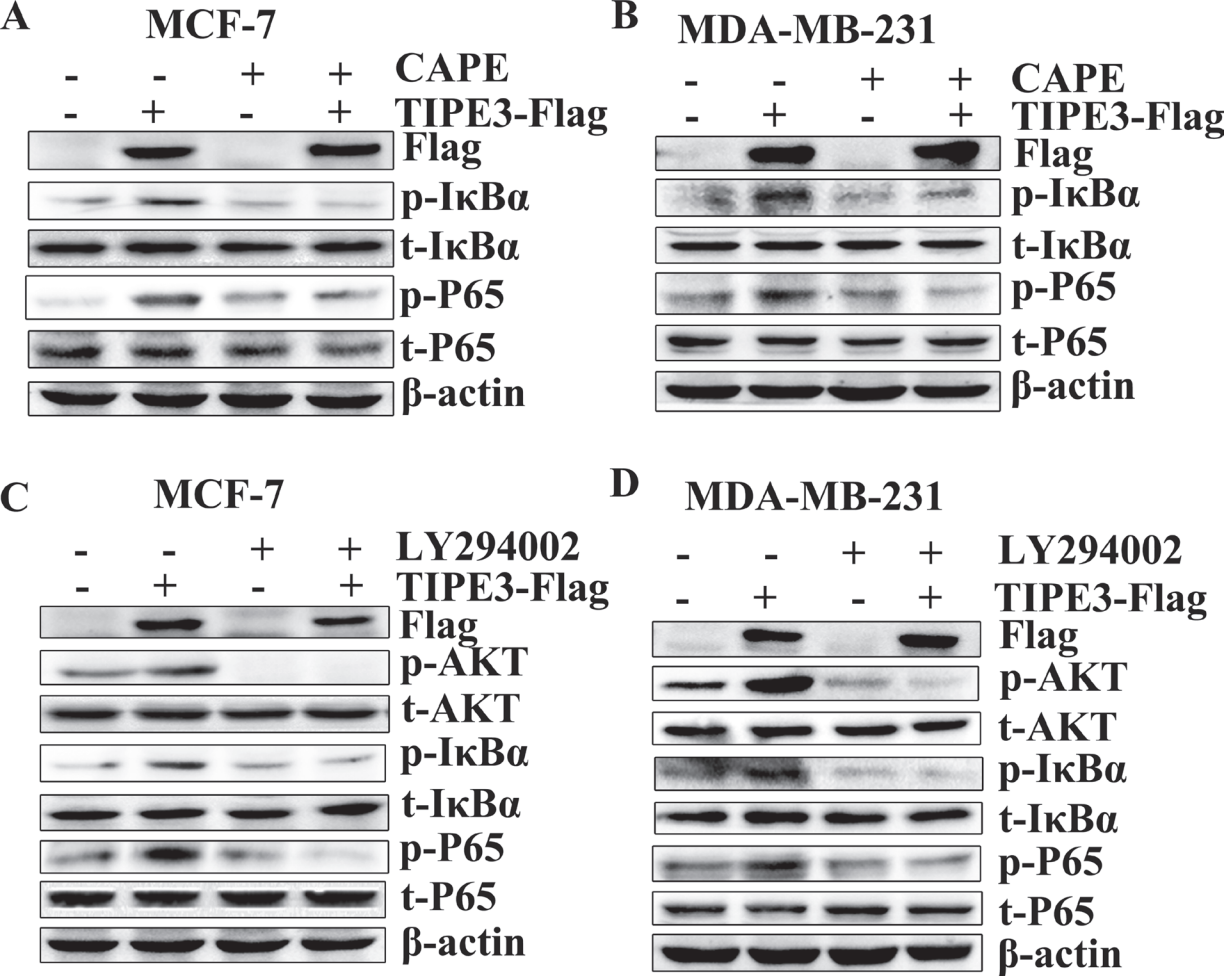
TIPE3-mediated activation of the NF-κB and AKT pathways in breast cancer cells Inhibiting NF-κB signaling by CAPE could block the activation of p-IkBa and p-P65 induced by overexpressed TIPE3 in MCF-7 (**A**) and MDAMB-231 cells (**B**). Blocking AKT signaling by LY294002 could downregulate the increased phosphorylation levels of p-AKT, p-IkBa and p-P65 induced by overexpressed TIPE3 in MCF-7 (**C**) and MDAMB-231 (**D**). All of the experiments were repeated at least three times with similar results and representative data are shown.

**Figure 8:**
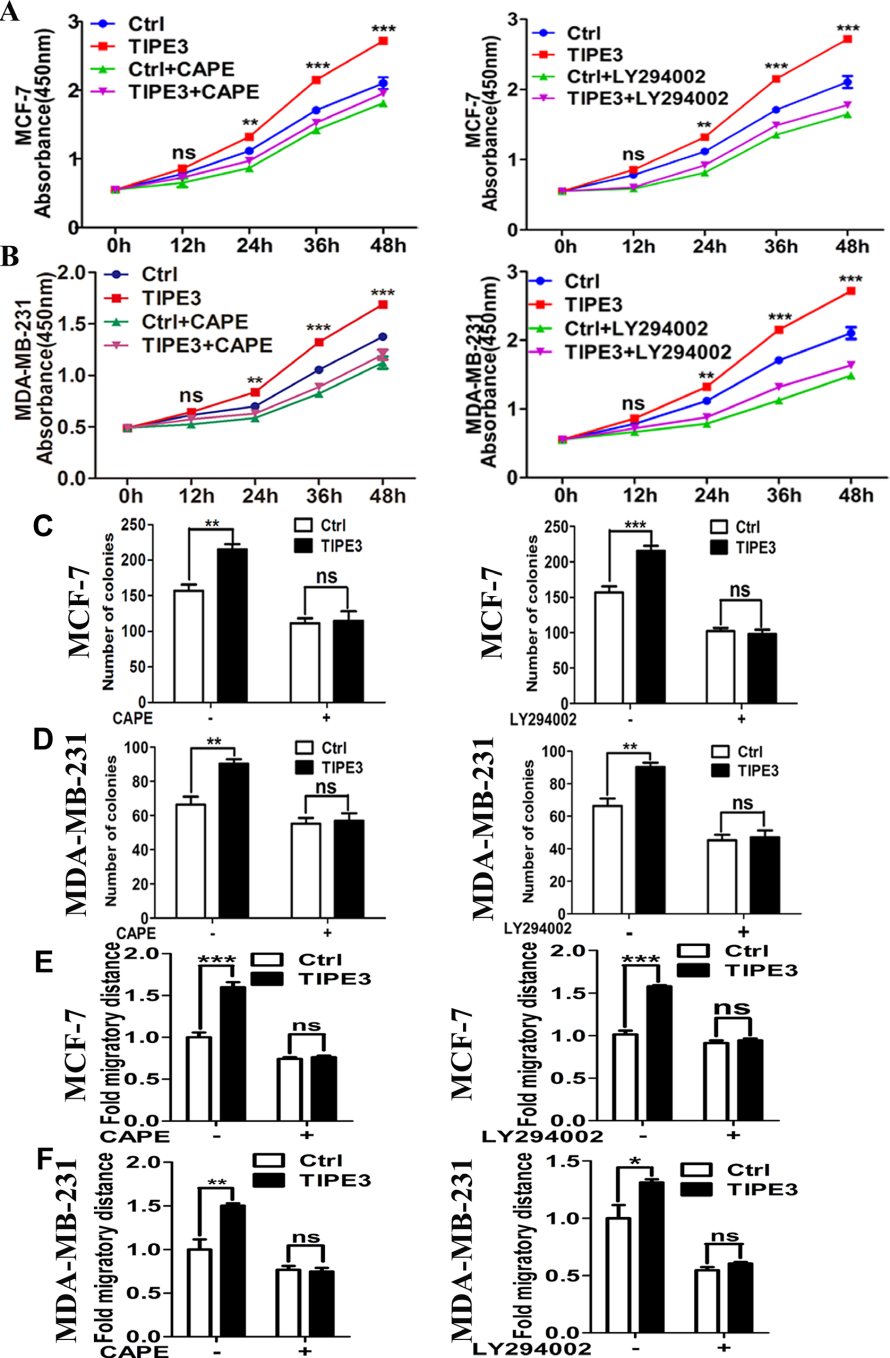
Inhibiting the NF-κB and AKT signaling blocks the TIPE3 effects on proliferation and migration of breast cancer cells CAPE or LY294002 could block the enhanced proliferation (**A**), colony formation (**C**), and migration (**E**) abilities induced by overexpressed TIPE3 in MCF-7 cells or MDA-MB-231 cells (**B**, **D** and **F**, respectively). All of the experiments were repeated at least three times with similar results and representative data are shown. (*P < 0.05, **P < 0.01 and ***P < 0.001).

To confirm the effect of TIPE3 on AKT and NF-κB pathways in breast cancer, inhibitors CAPE (inhibitor of NF-κB) [30] and LY294002 (inhibitor of PI3K inhibitor) [31] were used in this study. Results showed that the elevation of p-IkBα and p-P65 levels induced by overexpressed TIPE3 in MCF-7 and MDA-MB-231 cells was reduced markedly by CAPE (Figure 7A and 7B). Similarly, the activation of p-AKT induced by overexpressed TIPE3 was inhibited through blocking the AKT activity by LY294002 (Figure 7C and 7D). As a result, the enhanced proliferation (Figure 8A and 8B), colony formation (Figure 8C and 8D, Supplementary Figure 5A and 5B) and migration (Figure 8E and 8F, Supplementary Figure 5C and 5D) in MCF-7 and MDA-MB-231 cells induced by overexpressed TIPE3 were largely blocked by CAPE or LY294002. Interestingly, the elevation of p-IkBα and p-P65 induced by overexpressed TIPE3 was also largely blocked by LY294002 (Figure 7C and 7D), suggesting that TIPE3 might activate NF-κB pathway through AKT pathway in breast cancer cells [32, 33]. Taken together, these data demonstrate that TIPE3 promotes proliferation, migration and invasion of breast cancer cells by activating AKT and NF-κB pathways.

### TIPE3 promotes tumorigenesis and metastasis *in vivo*

To examine the functions of TIPE3 in tumorigenesis and metastasis *in vivo*, we established breast cancer subcutaneous xenograft and lung metastasis models in nude mice. In subcutaneous xenograft tumor models, we found that the tumors appeared more rapidly in nude mice injected with pRK5-TIPE3 plasmid than those in nude mice injected with pRK5- Ctrl vector (Table 3). The tumor of TIPE3 group grew more rapidly than Ctrl group (Figure 6A). As a result, the final tumor mean volume and weight of TIPE3 group was significantly increased compared with Ctrl group (Figure 6B–6D). The results from WB and IHC showed that TIPE3 protein expression was higher in tumor tissues with TIPE3 treatment (Figure 6E). In lung metastasis models, MDA-MB-231 cells infected with Lenti-shTIPE3 or shCtrl were injected into nude mice through the tail vein. After 50 days, the nude mice were killed and small numbers of white spots were observed on the lung surface. The HE staining results showed more and bigger metastatic foci in the lungs of nude mice injected with MDA-MB-231-shCtrl cells than in the lungs of nude mice injected with MDA-MB-231-shTIPE3 cells (Figure 6F). All these results indicate that TIPE3 promotes tumorigenesis and metastasis *in vivo*.

**Table 3:** TIPE3 protein promotes tumor formation in nude mice

Group	The number of nude mice appearing tumor (n)						
	5	6	7	8	9	10	(day)
pRK5	0	0	2	4	4	6	
TIPE3	1	3	6	7	7	7	

Note: When the diameter of tumor > 0.5 cm is considered as tumor formation.

## DISCUSSION

To our knowledge, this is the first report that TIPE3 plays a critical role in the carcinogenesis and progression of breast cancer. We found that TIPE3 was highly expressed in breast cancer tissues and positively correlated with HER2 expression. TIPE3 was able to promote significantly the proliferation of human breast cancer cells. More importantly, TIPE3 promoted the migration and invasion of human breast cancer by activating p-AKT and NF-κB pathways, and TIPE3 could accelerate the metastasis of human breast cancer cells in nude mice.

Previous research has showed that TIPE3 is highly expressed in most human carcinoma cell lines and promotes tumor malignant transformation [25, 26]. Breast cancer is the most frequently diagnosed cancer type in women. Tumor markers have been widely used for the diagnosis of breast cancer early stage assessment of the treatment response [34]. Our results showed that TIPE3 was upregulated markedly in malignant tissues of breast cancer compared to adjacent tissues from the same patients. Importantly, a positive correlation was found between the levels of TIPE3 expression and the pathological grading in breast cancer samples. Moreover, we found that the expression of TIPE3 was positively correlated with HER2 expression. Overexpression of HER2, Ki67 and p53 are found in 20∼30% of breast cancer patients and is associated with poor prognosis and relapse [35–38]. These results demonstrate that elevated expression of TIPE3 may be correlated with the increase of malignancy degree in breast cancer tissue and serve as a novel biomarker for the diagnosis of the earliest possible stage and response to therapy.

Firstly, we found that TIPE3 could promote the proliferation of breast cancer cells. The overexpression of TIPE3 in MCF-7 cells or MDA-MB-231 cells markedly increased cell growth and the size of colony formation in soft agar. Furthermore, overexpressed TIPE3 could reduce the population of G_0_/G_1_ cells and increase S/G_2_/M cells. These results confirmed the notion that TIPE3 could accelerate cell cycle progression [26]. Wherefore, knocking down the expression of TIPE3 in MDA-MB-231 cells inhibited cell growth, decreased the size of colony formation, and arrested cell cycle progression. Subcutaneous xenograft tumor experiment demonstrated that overexpressed TIPE3 could promote tumor formation *in vivo*. Importantly, the expression of TIPE3 was positively correlated with pathological grading and HER2 expression, suggesting that elevated expression of TIPE3 may be correlated with the increase of malignancy degree in breast cancer tissue. These data indicate that TIPE3 promotes breast cancer cells growth and further confirm the notion that TIPE3 may be a novel oncogene.

Furthermore, TIPE3 could accelerate breast cancer cell migration and invasion both *in vitro* and *in vivo*. Metastasis of breast cancer is a major cause of death for breast cancer patients [39]. PI3K-AKT signaling pathway regulates tumorigenesis and metastasis of breast cancer [40, 41] However, the role of TIPE3 in the migration and invasion of breast cancer has never been examined. We found that the levels of TIPE3 expression were higher in MDA-MB-231 and BT549 cells which were considered as more invasive cancer cells than MCF-7 and MDA-MB-468 cells [42], suggesting that TIPE3 might be associated with cancer migration and invasion. Overexpressed TIPE3 in MCF-7 cells could promote cell migration and invasion, while knocking down of TIPE3 in MDA-MB-231 cells reduced cell migration and invasion. Interestingly, the levels of TIPE3 staining in invasive ductal carcinoma were significant higher than that in DCIS from the same patients. Similarly, the staining of TIPE3 protein was stronger in lymph node metastatic breast cancer cells than that in DCIS or invasive ductal cancer cells from the same patients. Additionally, by using breast cancer lung metastasis model, we found that MDA-MB-231 cells treated with Lenti-siTIPE3 have greatly reduced ability to form metastatic foci in the lungs and livers than in shCtrl cells. These data suggested that TIPE3 expression might be associated with the progression and metastasis of human breast cancer.

Finally, TIPE3 regulates proliferation, migration and invasion in breast cancer cells by activating AKT and NF-κB pathways. Early studies have identified that TIPE3 promote tumorigenesis by activating PtdIns (3, 4, 5)P3 signaling pathway [26]. PI3K-AKT pathway plays an important role in tumorigenesis and promotes tumor malignant transformation including survival, proliferation and metabolism [43]. The PI3K/AKT signaling pathway is frequently activated in more than 50% of human breast cancers and most commonly through mutational activation of the PIK3CA gene [44]. Coordinately, PIK3CA mutations and AKT activation by phosphorylation (pAKT) are often identified at high frequencies in breast cancer [43]. In this study, we found that overexpression of TIPE3 in MCF-7 cells substantially upregulated p-AKT and p-IkBα phosphorylation and promoted p65 nuclear localization. Similarly, TIPE3 knockdown in MDA-MB-231 cells impaired AKT and IkBα activation. The inhibitors of AKT and NF-κB pathways, such as CAPE and LY294002, could block the enhanced proliferation, colony formation and migration of breast cancer cells induced by overexpressed TIPE3. Interestingly, blocking PI3K-AKT pathway by LY294002 largely abolished the activation of NF-κB induced by TIPE3 overexpression, suggesting that TIPE3 might activate NF-κB through AKT pathway during breast cancer development. This finding was consistent with studies that NF-κB cross-talks with AKT signaling [32, 33]. These results indicate that TIPE3 regulates proliferation, migration and invasion by activating AKT and NF-κB pathways. Studies suggested that genes, such as MMP2, MMP9, MMP11 and uPA, were found to be associated with breast cancer progression, angiogenesis and metastasis [45, 46]. MMP2, uPA were identified as indicator of tumor invasion and metastasis [47]. In this paper we found that the expression of MMP-2 and uPA was increased in TIPE3 overexpressed MCF-7 and MDA-MB-231 cells, while decreased in TIPE3 knocked down MDA-MB-231 cells. These results indicated that TIPE3 might promote breast cancer progression by upregulating the expression of MMP2 and uPA via activating AKT- NF-κB pathways, and might be an important indicator of breast cancer invasion and metastasis.

In summary, our findings demonstrated that TIPE3 protein was upregulated in breast cancer tissues and positively correlated to invasion and metastasis of human breast cancer cells by activating AKT and NF-κB signaling pathways. These results suggested that TIPE3 might serve as an indicator of breast cancer early stage diagnosis, invasion and metastasis and be a potential therapeutic target for breast cancer.

## MATERIALS AND METHODS

### Clinical specimens

A total of 96 human breast specimens, including tissue microarrays (BR961a, *n* = 48, purchased from Alenabiao, Xian, China) and breast tissues (*n* = 48) collected from patients who were admitted to the Shandong Qilu Hospital between February 2014 and July 2015, were used to detect the TIPE3 expression by immunohistochemistry. The diagnosis of breast cancer was made in accordance with the criteria approved by the Pathology and Genetics of Tumors of Breast of World Health Organization Classification of Tumors (2012, WHO). Among these samples, 25 specimens had adjacent breast tissues (10 ductal carcinoma *in situ* (DCIS) and 15 invasive ductal carcinoma), 24 specimens were diagnosed as invasive ductal carcinoma with ductal carcinoma *in situ* (DCIS), 62 cases were diagnosed as invasive ductal carcinoma with no DCIS. Among the invasive cancer, 36 cases had lymph node metastasis. Detailed clinical and pathologic information was provided by the manufacturers and Qilu hospital and summarized in Tables 1 and 2. Expression of ER, PR and HER2 are determined by immunohistochemical staining. All procedures were pre-approved by the Institutional Review Board (IRB) of the Shandong University.

### Cell culture

The breast cancer cell lines, MDA-MB-231, MCF-7, MDA-MB-468, SKBR3 and BT549 were purchased from Shanghai Cell Bank of Chinese Academy of Science (Shanghai, China). MDA-MB-231, BT549 and SKBR3 cell lines were grown in RPMI1640 medium (Hyclone, Massachusetts, USA) supplemented with 10% FBS (Gibco-BRL) at 37°C in a 5% CO_2_ atmosphere. MDA-MB-468 and MCF-7 were grown in DMEM medium (Gibco) supplemented with 10% FBS (Gibco) at 37°C in a 5% CO_2_ atmosphere.

### Plasmid transfection and viral infection

Expression plasmid pRK5-hTIPE3 with a Flag tag at the C-terminus was constructed by Invitrogen Co. MCF-7 and MDA-MB-231 cells were seeded on six-well plate (Corning) and transiently transfected with pRK5-hTIPE3 (marked as MCF-7-hTIPE3 and MDA-MB-231-TIPE3 respectively), or transfected with pRK5 vector as controls, using Lipofectamine 2000 according to the manufacturer's protocol (Invitrogen, Carlsbad, CA, USA). Cells were then treated with the PI3K inhibitor LY294002 (10 mM, Apexbiotochnology, USA) or the NF-κB inhibitor CAPE (70 mM, Apexbiotochnology, USA). In addition, MDA-MB-231 cells with high expression of TIPE3 were infected with lentiviruses encoding control shRNA (shCtrl) or TIPE3-specific shRNA (shTIPE3). The viruses were constructed, packed, and purified by GeneChem Co. (Shanghai, China). The control and target sequences are shCtrl: TTCTCCGAACGTGTCACGT, shTIPE3-1: TAGATGAGAAAGTCCTTTA, shTIPE3-2: ACAAGATCATGAAAGACTT and shTIPE3-3: GCTCTACAAAGTCACCAAA. We performed the infection experiment according to the protocol provided by the manufacturer.

### Quantitative real-time PCR (qRT-RCR)

Total RNA was extracted from breast cancer cells using RNAfast200 (Fastagen, Shanghai, China) or Trizol (Invitrogen) and reversely transcribed into cDNAusing PrimeScriptTM RT reagent Kit (TaKaRa, Japan), according to the manufacturer's instructions. QRT-PCR was performed as described previously [15]. The primers were designed by PrimerBank and synthesized by (BGI-Tech, Shenzhen, China). The sequences of primers were as follows: hTIPE3: Forward 5′-TTCAGAGGGGAAAGGGACT-3′, reverse 5′-AACATCAGGACCTGCGGC-3′ [25]; h-β-actin: Forward 5′-AGTTGCGTTACACCCTTTC-3′, reverse 5′-CCTTCACCGTTCCAGTTT-3′; h-uPA: Forward 5′-CACGCTTGCTCACCACAACGACA-3′, reverse 5′-CTGACACTCCCGGTGGGAAATCA-3′ [13]; h- MMP2: Forward 5′-GATACCCCTTTGACGGTAA GGA-3′, reverse5′-CCTTCTCCCAAGGTCCATAGC-3′. Quantitative PCR (qPCR) was performed using iQSybr Green Supermix (Bio-Rad). cDNA levels were determined using a standard curve and normalized to β-actin. Each sample was run in triplicates. A melting-curve analysis was performed to ensure the specificity of the products.

### Western blot

Protein was collected from the supernatant of breast cancer cells lysed on ice in RIPA lysis buffer (Beyotime, Shanghai, Chain) containing 1% protease and 0.1% phosphatase inhibitors. Western blot was performed as described previously [14]. Specific primary antibodies include anti-hTIPE3 (1:1000, Sigma, USA) and anti-flag (1:1000, Sigma, USA), anti-pAKT and anti-AKT, anti-IkBα and anti-pIkBα, anti-p-P65 and anti-P65 antibodies (1:1000, Cell Signaling Technology, Beverly, USA), anti-β-actin (1:1000, ZSGB-BIO), anti-β-tubulin antibody and anti-LaminA antibody (Sino Biological Inc, China).

### Immunohistochemistry (IHC)

Human breast tissue microarrays and cancer tissues were assessed for TIPE3 expression using immunostaining, as previously described [25]. Specific primary antibodies were prepared for anti-hTIPE3 (1:1000, Sigma, USA). Secondary staining was performed using HRP-conjugated goat anti-rabbit IgG mAb (PV-6001, ZSGB-BIO, Beijing, China) and the reaction developed with Histostain Bulk Kit and DAB kit (ZLI-9018, ZSGB-BIO, Beijing, China). Sections were then mounted in neutral balsam (Invitrogen) and examined with an optical microscope (Olympus BX51, Olympus; Tokyo, Japan). All slides were independently analyzed by two pathologists in a blinded manner, and scored based on both intensity of TIPE3 expression and the percentage of TIPE3 positive cells as follows. The scores were evaluated basing on the intensity of TIPE3 expression: 0/-, no staining; 1/+, weak staining; 2/++, moderate staining; 3/+++, strong staining. The percentage of TIPE3 positive neoplastic immunoreactive cells:0, < 1%; 1, 1–20%; 2, 21–40%; 3, 41–60%; 4, 61%∼80%; 5, ≥ 81%; The two scores for each slide were combined to produce a final grade of TIPE3 expression [26]. When there were discrepancies between the two pathologists, the average score was used.

### Cell proliferation and colony formation assay

For the cell proliferation assay, 1 × 10^4^ TIPE3-overexpressing MCF-7-TIPE3 or MDA-MB-231-TIPE3 cells, and MDA-MB-231-shTIPE3 cells and their controls were treated with or without CAPE or LY294002, seeded and cultured in 96-well plates. Cell proliferation was evaluated at different time intervals by Cell Counting Kit 8 (CCK8, Dojindo, Tokyo, Japan) according to its protocol, including 0 h, 24 h, 48 h, 72 h and 96 h. The optical density (OD) value was measured at 450 nm to calculate the numbers of viable cells in each well on a microplate reader (Model 680, BIO-RAD).

For the colony formation assay, 1 × 10^3^ TIPE3-overexpressing MCF-7-TIPE3 or 3 × 10^3^ MDA-MB-231-TIPE3 cells treated with or without CAPE or LY294002 were seeded and cultured in six-well plates for 1–2 weeks until visible colonies were formed. Survival colonies were fixed with 100% methanol, stained with 1% of crystal violet (DAMAO REAGENT, Tianjin, Chain). Colonies containing more than 100 cells were counted. The experiments were performed in triplicate.

### Flow cytometry for cell cycle

For the cell cycle assay, MCF-7-Ctrl and MCF-7-TIPE3 cells or MDA-MB-231-shCtrl and MDA-MB-231-shTIPE3 cells were cultured in 6-well plates for 48 h. Then cells were collected to fix in ice-cold 75% ethyl alcohol at 4°C overnight and stained with propidium iodide (PI) at 4°C for 30 min. PI incorporation was analyzed by flow cytometry for DNA content and data were analyzed with Cytomics FC500 (Beckman Coulter, Pasadena, CA, USA).

### Cell migration and invasion

Migration ability of breast cancer cells was analyzed by wound healing and transwell migratory assay. For wound healing assay, MCF-7-TIPE3 cells or MDA-MB-231-TIPE3 and their controls treated with or without CAPE or LY294002, or MDA-MB-231-shCtrl and MDA-MB-231-shTIPE3 cells were cultured in 6-well plates. Then a wound of cell mono-layers was carefully wounded with a sterile 200 μl pipette tip. After washing twice with PBS, cells were incubated in growth medium containing 1% FBS for 48 h. Then images of cell wound were captured at 0 h and 48 h using a light microscope. Distance of cell migration was determined by measuring width of the wound and calculated using the width difference from treated breast cancer cells divided by that from mock control cells.

For migration assay, cells were suspended in serum-free medium and seeded into the upper chamber of a Transwell apparatus (24-well insert, 8 μm pore size; Corning, USA). 600 mL medium containing 10% FBS was added to the lower chambers. For invasion assays, the membranes were precoated with 50 mL 1:3 mixture of BD Matrigel (BD Biosciences, USA) and serum-free medium for 4 h under sterile conditions. After 12 h (migration assays) or 36 h (invasion assays), the cells migrated to the bottom surface of the membrane were fixed with 100% methanol, stained with 1% of crystal violet (DAMAO REAGENT, Tianjin, Chain). The migration and invasion ability was examined by counting the number of migrated cells in 5 random fields by microscopy with 100× magnification. The experiments were repeated at least three times.

### Tumor growth and metastasis studies *in vivo*

7-week-old female BALB/c athymic nude mice were purchased from Weitonglihua Co. (Beijing, China) and housed in under specific pathogen-free (SPF) conditions. The nude mice and experiment protocols were approved by the Institutional Animal Care and Use Committee of Shandong University. For subcutaneous inoculation, MCF-7 cells (1 × 10^6^) in 100 mL PBS were injected subcutaneously into the left flank of the hind legs. The mice were randomly divided into two groups (*n* = 7). In 24 h postinjection, 20 μg pRK5-TIPE3 plas mid or pRK5 - Ctrl vector were injected subcutaneously into the same parts injecting cells. Appearance of tumors were assessed according to the length (d > 0.5 cm) of the tumor every day. Formed tumors were injected with 10 μg of pRK5-TIPE3 plasmid or empty pRK5-Ctrl vector at the 15th day. The tumors were measured every 3 days and the tumor volume was calculated by the formula length × width^2^/2 [48]. The mice were killed 25 days after the inoculation. Numbers of mice appearing tumor volume of different groups were analyzed. For experiment of metastasis, MDA-MB-231 cells (1 × 10^6^) infected with virus-mediated shCtrl or shTIPE3 in 100 mL PBS were injected into tail veins of nude mice (*n* = 5). The mice were killed 50 days after the inoculation. The visible lung surface macrometastatic lesions appeared as white spots were counted and evaluated pathologically by HE staining on sections from embedded samples.

### Statistical analysis

The results are expressed as the mean ± SD. The correlations between clinical pathological features and expression of TIPE3 were analyzed using Spearman's rank correlation test (SPSS 16.0, Inc, USA). Unpaired *t-test* was used to compare between different groups. A value of *p* < 0.05 was considered statistically significant.

## SUPPLEMENTARY MATERIALS FIGURES AND TABLES


