# Supplementary material for: TIPE3 protein promotes breast cancer metastasis through activating AKT and NF-κB signaling pathways
Source: Oncotarget. 2017 Mar 23;8(30):48889–904. doi: 10.18632/oncotarget.16522 (PMC5564733; doi:10.18632/oncotarget.16522)
Supplement: Supplementary file 1 [file oncotarget-08-48889-s001.pdf]

TIPE3 protein promotes breast cancer metastasis through activating AKT and NF-κB signaling pathways

Supplementary Materials

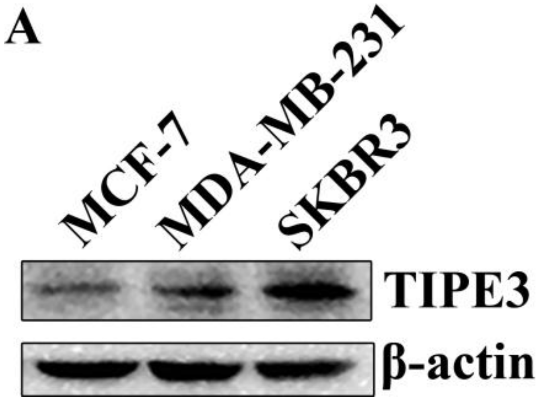

Supplementary Figure 1: TIPE3 expression in cell lines. The level of TIPE3 protein in HER2-positive breast cancer cells (SKBR3) was much higher than that in HER2-negative breast cancer cells (MCF-7 and MDA-MB-231) by Western blot. All of experiments were repeated at least three times with similar results and representative data are shown.

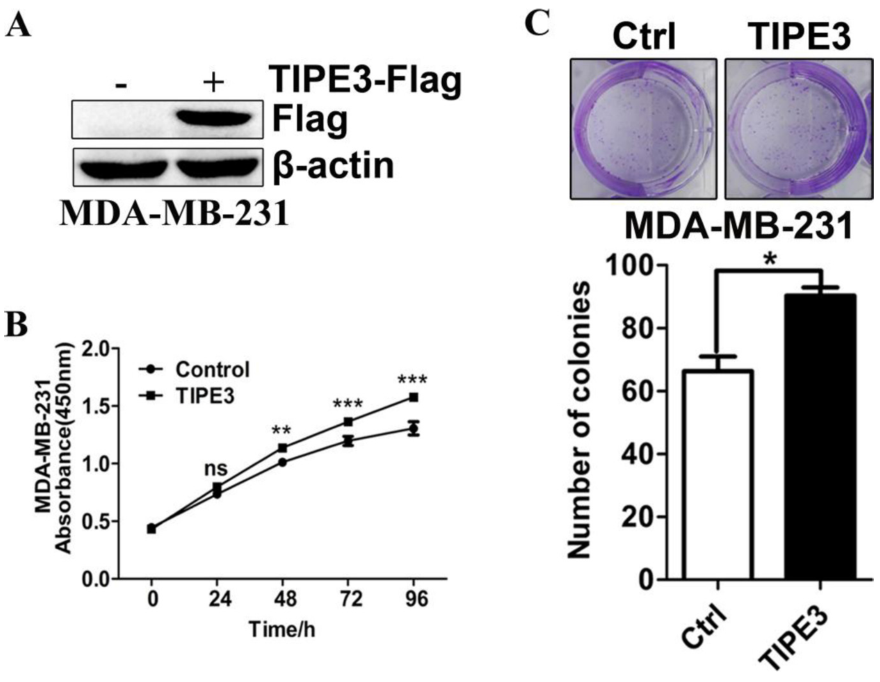

Supplementary Figure 2: Overexpression of TIPE3 promoted proliferation and colony formation of MDA-MB-231 cells. Overexpressed TIPE3 in MDA-MB-231 cells were examined using western blot (**A**), and promoted cells proliferation (**B**) and colony formation (**C**). All of the experiments were repeated at least three times with similar results and representative data are shown. (*$P < 0.05$, **$P < 0.01$ and ***$P < 0.001$).

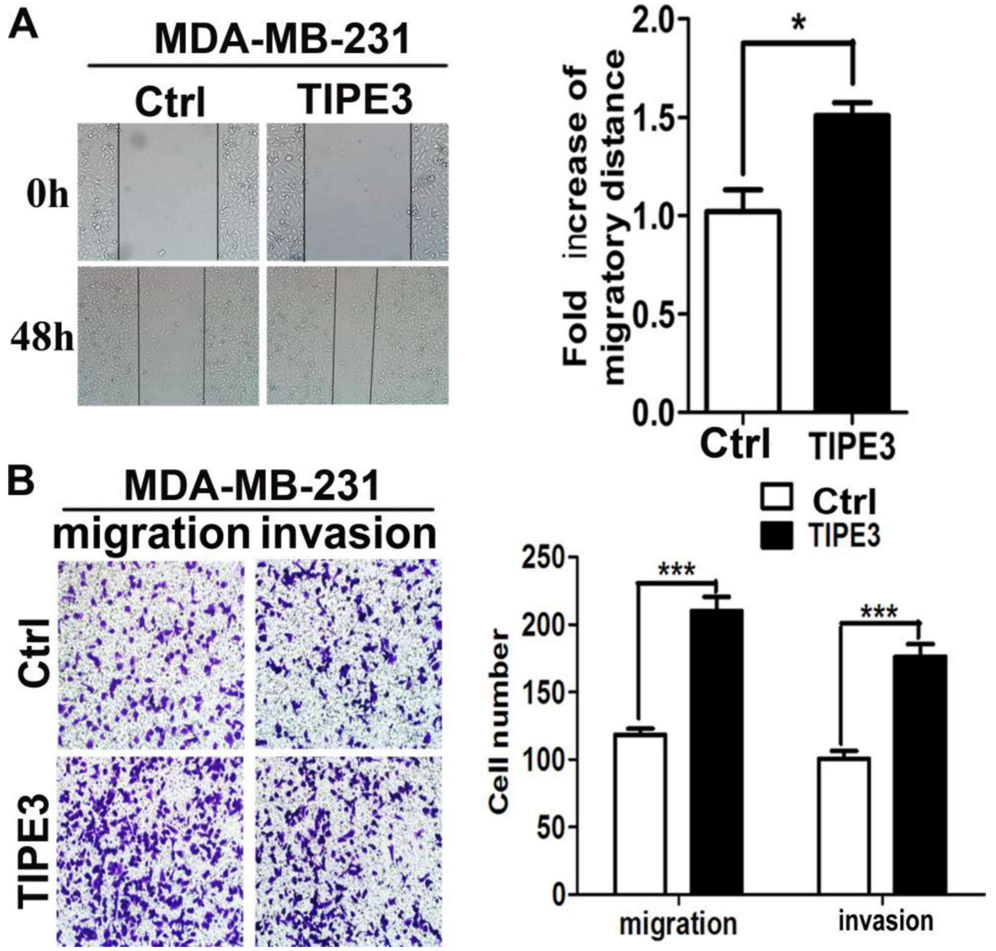

Supplementary Figure 3: TIPE3 promoted migration and invasion of MDA-MB-231 cells. MDA-MB-231-shTIPE3 cells or Ctrl cells were subjected to wound healing assay (**A**), transwell migration (**B**) and matrigel invasion assays (B). All of the experiments were repeated at least three times with similar results and representative data are shown. (*$P < 0.05$, **$P < 0.01$ and ***$P < 0.001$).

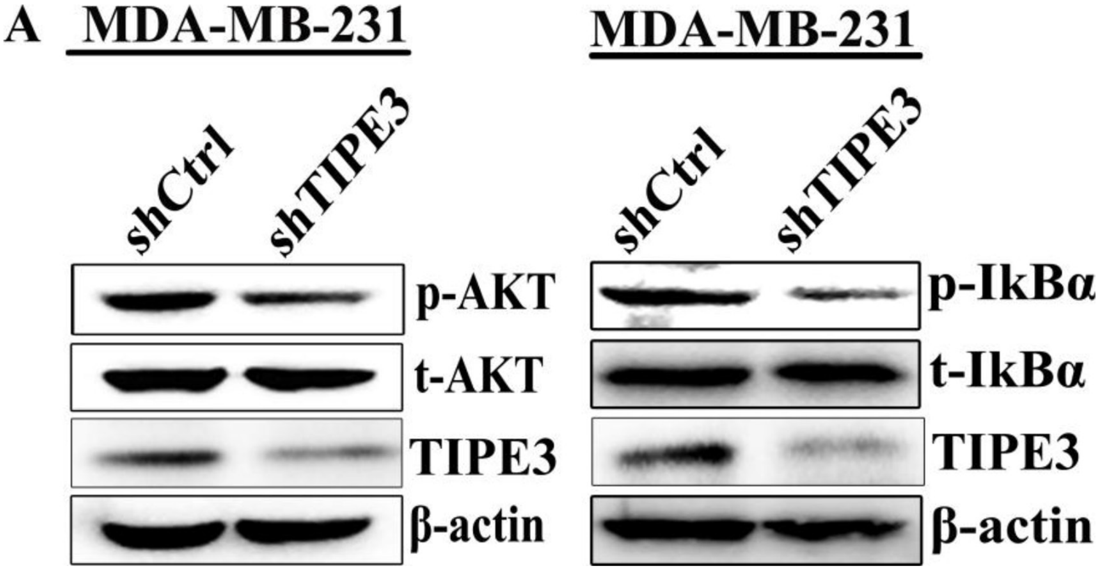

Supplementary Figure 4: Knocking down TIPE3 in MDA-MB-231 cells prevented the activation of p-AKT and p-IκBα. The levels of p-AKT and p-IκBα were decreased in MDA-MB-231-shTIPE3 cells.

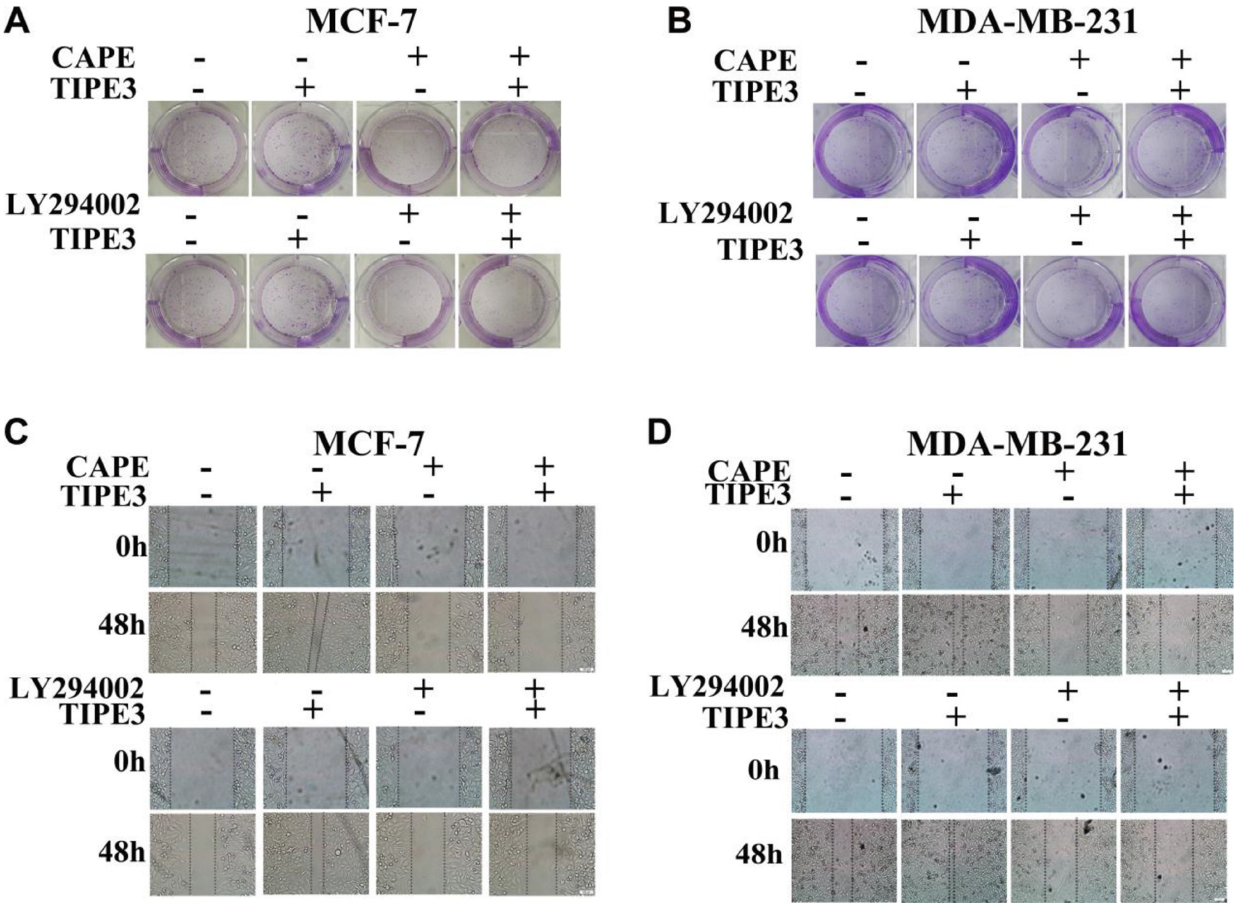

Supplementary Figure 5: The enhanced colony formation and migration ability induced by overexpressed TIPE3 in breast cancer cells were abolished through blocking the NF-κB and AKT signaling with CAPE or LY294002. The enhanced clonogenicity induced by overexpressed TIPE3 in MCF-7 cells (**A**) or MDA-MB-231 cells (**B**) were blocked using CAPE or LY294002. The enhanced migration ability induced by overexpressed TIPE3 in MCF-7 cells (**C**) or MDA-MB-231 cells (**D**) wre blocked using CAPE or LY294002. All of the experiments were repeated at least three times with similar results and representative data are shown.

Supplementary Table 1: Expression scores of TIPE3 protein in breast cancers

Clinical pathological features	Number of cases	Expression scores of TIPE3								*P*-value*
		**0**	**2**	**3**	**4**	**5**	**6**	**7**	**8**	
Adjacent tissues*	25	10	5	6	3	1	0	0	0	< 0.0001*
DCIS*	34	1	2	7	9	6	4	5	0	0.0033
Invasive cancer	86	1	4	8	14	13	15	17	14	

Note: P-value*: unpaired *t*-test.
P < 0.0001*, TIPE3 protein in Adjacent tissues vs TIPE3 in Cancer tissues.
P = 0.0033, TIPE3 protein in DCIS tissues vs TIPE3 in invasive tissues.

Supplementary Table 2: Comparison of TIPE3 expression scores between invasive breast cancer and lymph node metastatic tissues from same patients

Clinical pathological features	Number of cases	Expression scores of TIPE3								*P*-value*
		**0**	**2**	**3**	**4**	**5**	**6**	**7**	**8**	
Invasive cancer*	36	0	2	4	6	5	5	7	7	0.0276*
Lymphatic met cancer*	36	0	1	1	4	3	4	10	13	

Note: P-value*: unpaired *t*-test.
Invasive cancer*: invasive cancer with lymphnode metastasis.
P = 0.0276*, TIPE3 protein in lymphatic metastasis carcinoma vs in invasive ductal carcinoma.
